# Protocol for a systematic review and meta-analysis on Janus kinase inhibitors in the management of vitiligo

**DOI:** 10.1186/s13643-024-02522-3

**Published:** 2024-04-19

**Authors:** Shelly Pranić, Anika Pulumati, Dubravka Vuković

**Affiliations:** 1 School of Medicine, University of Split, Šoltanska 2, 21000 Split, Croatia; 2 Cochrane Croatia, Šoltanska 2, 21000 Split, Croatia; 3 University of Missouri-Kansas City School of Medicine, 2411 Holmes, Kansas City, MO 64108 USA; 4 University Hospital of Split, Spinčićeva 1, 21000 Split, Croatia

**Keywords:** Vitiligo, Janus kinase, Randomized controlled trials, Observational studies, Systematic review, Meta-analysis

## Abstract

**Background:**

Vitiligo is a disease that affects people of all skin shades and can impact their quality of life. Reliable evidence on the effectiveness and adverse events associated with the recent use of Janus kinase (JAK) inhibitors to treat vitiligo is needed. This protocol for a systematic review and meta-analysis seeks to collect evidence from both randomized controlled trials (RCTs) and observational studies to determine the effectiveness and patient-centered outcomes concerning treatment with JAK inhibitors.

**Methods:**

We will conduct a systematic review of the literature for RCTs and observational studies that used upadacitinib, ritlecitinib, brepocitinib, ifidancitinib, cerdulatinib, deglocitinib, baricitinib, tofacitinib, and ruxolitinib JAK inhibitors as treatments for vitiligo compared to placebo, no treatment, or combination therapies. We will systematically search from inception in Epistemonikos, MEDLINE, Scopus, Cochrane Central Register of Controlled Trials, EMBASE, ClinicalTrials.gov, PsycINFO, Allied and Complementary Medicine Database, Latin American and Caribbean Health Sciences Literature, Web of Science Core Collection, relevant preprint servers, and the gray literature. Ethics approval was not sought as the protocol and systematic review will not involve human participants, but rather summarized and anonymous data from studies. Primary outcomes include quality of life, percentage repigmentation, decreased vitiligo within 1 year or more, lasting repigmentation after a 2-year follow-up, cosmetic acceptability of repigmentation and tolerability or burden of treatment, and adverse events. Secondary outcomes are patient and study characteristics. We will include full-text articles, preprints, and clinical trial data in any language and all geographic regions. For data sources unavailable in English, we will obtain translations from global collaborators via the Cochrane Engage network. We will exclude articles for which sufficient information cannot be obtained from the authors of articles and systematic reviews. At least two investigators will independently assess articles for inclusion and extract data; reliability will be assessed before subsequent selection and data extraction of remaining studies. The risk of bias and certainty of evidence with Grading of Recommendations Assessment, Development, and Evaluation guidelines will be assessed independently by at least two investigators. We will estimate treatment effects by random-effects meta-analyses and assess heterogeneity using *I*^2^. Data that cannot be included in the meta-analysis will be reported narratively using themes.

**Discussion:**

The proposed systematic review and meta-analysis describe the methods for summarizing and synthesizing the evidence on the effectiveness and patient-centered outcomes concerning the treatment of vitiligo with JAK inhibitors that were recently approved for this indication. To disseminate further the results of our systematic review, we plan to present them at international conferences and meetings. Our findings will provide robust evidence to facilitate decision-making at the policy or practitioner level.

**Systematic review registration:**

PROSPERO CRD42023383920.

**Supplementary Information:**

The online version contains supplementary material available at 10.1186/s13643-024-02522-3.

## Introduction

Vitiligo is a chronic auto-immune skin disorder caused by the destruction of melanocytes resulting in white patches on the skin and hair in all races where its worldwide prevalence in adults varies from 0.5 to 2% ADDIN EN.CITE [1, 2]. Predominantly, younger people under the age of 20 years are affected [1, 2]. Of the 0 to 2% prevalence in children with vitiligo worldwide, evidence suggests that 25% or more of pediatric cases occur up to 10 years of age [3]. However, the prevalence may differ depending on whether its occurrence is reported to healthcare providers, which may be more likely in regions with dark-skinned people such as in Africa, Asia, and Latin America (including Mexico and Brazil) [1]. Consequently, the prevalence of vitiligo may indeed depend on the geographic region [3]. Studies from Benin, Togo, Senegal, Nigeria, Tanzania, and other African countries showed a prevalence of vitiligo ranging from 0.13 to 2.8% [4–9]. Lu and colleagues reported a 0.1% prevalence in the Chinese population [10]. A study from India reported an 8.8% prevalence of vitiligo [11]. Further, the prevalence of vitiligo in adults ranged from 0.21 to 4% in Mexico and 0.04 to 0.54% in Brazil [12, 13].

Research is ongoing related to the cause of vitiligo, but findings suggest family history, autoimmunity, or extrinsic factors alone or in combination may play a role [14–17]. Patients usually do not report symptoms associated with vitiligo, but Ezzedine et al. found that almost 20% of patients reported itchiness at the site of a new lesion [1, 18]. Most commonly, vitiligo is found near the orifices, the face, and the upper and lower extremities. The two types of vitiligo depend on the regions of the body affected [1, 18]. Non-segmental vitiligo is the most common type, also known as vitiligo vulgaris; is symmetrical; and may spread from affected regions, while segmental vitiligo affects one side of the body and is unlikely to spread to other body regions [1, 18].

Psychological and social consequences including anxiety and stigmatization can affect the quality of life of affected individuals; especially those with darker skin shades [19–21]. Specifically, previous studies found that individuals with vitiligo felt stigmatized and experienced low quality of life due to perceiving the visibility of their affected skin parts as socially unacceptable [19, 22, 23]. The quality of life assessed with the vitiligo quality of life (VitiQoL) questionnaire was poor in a sample of adult Nigerian patients with vitiligo [23]. In a cross-sectional study of adults and children with vitiligo in Brazil, the participants reported that mostly stigma from the disease affected their quality of life [22].

Various treatment options are available for vitiligo. A 2015 systematic review [1] described therapies for vitiligo including topical treatments: (1) topical corticosteroids, (2) intralesional corticosteroids, (3) topical vitamin D analogues, (4) topical calcineurin inhibitors, (5) khellin, (6) pseudocatalase and catalase/dismutase superoxide, (7) melagenina (human placental extract), (8) tetrahydrocurcuminoid cream, (9) topical anti-oxidant gel; oral therapies: (10) psoralen and ultraviolet A (UVA); light therapies: (11) punch grafts, minigrafts, and skin thickness grafts, (12) melanocyte transplantation, (13) fractional carbon dioxide (CO2) laser, (14) psychological therapy, and (15) complementary therapies. In 2022, the US Food and Drug Administration, followed by the European Medicines Agency in 2023, approved the topical use of the Janus kinase inhibitor (JAK) ruxolitinib for the treatment of non-segmental vitiligo in individuals 12 years of age and older [24]. Other JAK kinases including upadacitinib, ritlecitinib, brepocitinib, ifidancitinib, cerdulatinib, deglocitinib, baricitinib, and tofacitinib have not been approved for the treatment of vitiligo. In vitiligo, the JAK kinase/signal transducer and activator of transcription (STAT) pathway is activated by interferon (IFN)-gamma-chemokine produced from melanocyte-specified cluster of differentiation 8 (CD8 +)T cells [25]. JAK kinases subsequently phosphorylate STATs that translocate to the nucleus to activate IFN-gamma-inducible genes. Interest in inhibitors of the JAK/STAT pathway has recently increased as a target for vitiligo therapy as this pathway modulates immune cell activation after response to cytokines [25]. Due to the novel application of JAK inhibitors to treat vitiligo, systematic reviews are needed to summarize comprehensively adverse events and patient-centered outcomes surrounding the new treatments.

A recent systematic review and meta-analysis of observational studies and clinical trials by Phan and colleagues described that robust evidence is needed to determine the effectiveness of Janus kinase (JAK) inhibitors in treating vitiligo [26]. However, this systematic review lacked an assessment of patient-centered outcomes, which are of utmost importance surrounding a potentially stigmatizing disease such as vitiligo. The authors did not include an internationally agreed-upon core set of outcomes for vitiligo including tolerability of treatments and cosmetic acceptability of repigmentation [27]. Phan and colleagues also lacked an assessment of the quality of the individual studies that comprised the review, which precludes the weighing of the strength of the evidence against the results. Similarly, a 2023 meta-analysis of JAK inhibitors for the treatment of vitiligo also lacked an assessment of the quality and risk of bias of the evidence and patient-centered outcomes [28]. Additionally, the emerging off-label use of JAK inhibitors for vitiligo warrants further investigation. Thus, we plan to perform an updated systematic review and meta-analysis with an assessment of the quality and risk of bias that will describe patient-centered outcomes deemed important by patients and clinicians and the effectiveness of the most recent available JAK1, JAK2, and JAK 3 inhibitors to treat vitiligo including JAK 1/2 inhibitors ruxolitinib and baricitinib, JAK1/3 inhibitors upadacitinib, tofacitinib, ifidancitinib, and JAK1-3 inhibitor deglocitinib, JAK and spleen tyrosine kinase inhibitor cerdulatinib, JAK 1 and tyrosine kinase 2 inhibitor brepocitinib, and JAK3 and the tyrosine kinase expressed in hepatocellular carcinoma (TEC) kinase family inhibitor ritlecitinib for the treatment of non-segmental and segmental vitiligo [29–32]. Our Patient, Intervention, Comparator, and Outcome (PICO) (refer to Table 1 Table  review question is, “In children and adults with vitiligo, are topical or systemic JAK inhibitors at various dosages more effective than standard therapy for skin repigmentation within 1 year or longer?”.

**Table 1:** Patient, Intervention, Comparator, and Outcome (PICO) criteria concerning Janus kinase (JAK) inhibitors for the treatment of vitiligo

Patient	Intervention (mode of administration)	Intervention (dose and dose frequency studied)	Intervention (duration of therapy)	Comparator	Outcome measures*	Study type
Children and adults with vitiligo	Topical or systemic Janus kinase (JAK) inhibitors	Various doses and frequencies	Within 1 year or longer	Standard therapy	Quality of life	Observational or interventional
					Percentage of repigmentation	
					Cosmetic acceptability of repigmentation	
					Decreased spreading of the disease	
					Lasting repigmentation due to treatment after a 2-year follow-up	
					Tolerability or burden of treatment	
					Adverse events	

*Core outcome set for vitiligo clinical trials obtained from Eleftheriadou et al. [27]

## Outcomes

### Primary outcomes

The following are the primary outcomes:

1. Quality of life using a validated tool (e.g., Vitiligo Quality of Life Index (VitiQoL), Dermatology Quality of Life Index (DLQI), Children’s Dermatology Quality of Life Index (CDLQI), or Skindex-29)
2. Percentage of repigmentation
3. Cosmetic acceptability of repigmentation
4. Decreased spreading of the disease (decreased size of lesions or patches of depigmented skin due to treatment) within 1 year or more than 1 year
5. Lasting repigmentation due to treatment after a 2-year follow-up
6. Tolerability or burden of treatment
7. Adverse events

### Secondary outcome

The secondary outcome is characteristics of the patients and included studies.

## Methods

### Sources of information and literature search

The systematic review protocol has been registered in the PROSPERO database (CRD42023383920). Ethics approval was not sought as the protocol and systematic review will not involve human participants, but rather summarized and anonymous data from studies. We followed the guidance provided by the Preferred Reporting Items for Systematic Reviews and Meta-Analyses Protocols (PRISMA-P) statement [33] (refer to the checklist in Additional file 1) and will follow guidelines for reporting systematic reviews according to the PRISMA 2020 statement [34]. We consulted with a medical librarian and a dermatologist to choose the Medical Subject Headings search terms for observational and interventional studies on JAK inhibitors for the treatment of vitiligo. Additional search terms or the review question will be revised if needed that pertain to our current evaluation. We will systematically search Epistemonikos, MEDLINE (Ovid), Scopus, Cochrane Central Register of Controlled Trials (CENTRAL) [Cochrane Library], ClinicalTrials.gov, PsycINFO (Ovid), Allied and Complementary Medicine Database (AMED) [Ovid], Latin American and Caribbean Health Sciences Literature (LILACS), and Web of Science Core Collection from inception to the present. Relevant preprint servers (e.g., BioArxiv, MedArxiv) and the gray literature will also be searched. Study authors will be contacted for further information. Additional file 2 shows the keywords intended for the search and a draft search strategy for Web of Science, Scopus, and MEDLINE (Ovid). Additional file 3 shows the pilot search results obtained on 27/01/24.

### Study eligibility criteria

#### Inclusion and exclusion criteria

We will include full-text randomized controlled trials (RCTs) and non-randomized studies (observational studies). We used the Population, Intervention, Comparison, and Outcome, and Study type (PICOS) approach to identify the studies to be included. The population includes RCTs and observational studies should include JAK inhibitors as an intervention compared to a type of standard/usual care, placebo, nothing, or combined treatments of any form to assess the effectiveness of the treatment of vitiligo as the outcome. The patient population will include adults and children. We will include full-text articles, preprints, and clinical trial data in any language and all geographic regions without time restrictions. Sources up until the date of search will be included and any updated searches to include new publications will be described.

We will exclude (1) articles without sufficient descriptions or results about the intervention (protocols, conference proceedings, abstracts, letters, editorials, or commentary) even after contacting study authors and (2) systematic reviews with or without meta-analyses.

#### Risk of bias assessment

The assessment of risk of bias of each article will be assessed by using adapted criteria from the Cochrane Library Guidelines [35, 36] or the Risk of Bias (RoB 2) tool [37] independently by at least two investigators. The article quality will be categorized as methodologically strong or weak based on the (1) study design (e.g., randomized controlled trials will be deemed strong, while cross-sectional studies will be deemed as weak), (2) sample size (> 150 participants indicated strong; < 50 participants, weak), (3) ascertainment of repigmentation (visually by a clinician using photographs or planimetry; more objective methods will be deemed strong), (4) representativeness of the sample (≥ 2 institutions indicated strong, < 2 institutions will be deemed as weak), (5) descriptive characteristics of participants (reported data on sex, age, race, duration of vitiligo, and educational level indicated strong; missing information on sex, age, race, or educational level, weak), (6) method generation of the randomization sequence, (7) method of allocation concealment, (8) blinding involved, (9) loss to follow-up, (10) aims and interventions (doses, treatment duration), and (11) whether or not compliance to treatment was reported. Cutoff scores for the sample size, representativeness, and descriptive characteristics will be based on thresholds used in a previous systematic review of vitiligo interventions [1]. If at least one of the categories will be rated as high, the trial will be considered as high risk of bias. If all the domains are rated as low risk of bias, then the trial will be rated as low risk of bias. If unrated as either high or low risk of bias, then we will label the trial with an unclear risk of bias. At least two reviewers will perform data extraction and risk-of-bias assessment independently. We will discuss unclear eligibility among the reviewers until consensus (with an overall agreement of Cohen *κ* ≥ 80%) is reached for those cases with unclear eligibility.

#### Certainty of the evidence assessment

We will assess the level of certainty regarding the risk of bias, imprecision, inconsistency, indirectness, and publication of each of the important patient-centered primary outcomes across studies (as described above in the Outcomes section) using the Grading of Recommendations Assessment, Development, and Evaluation guidelines (GRADE) [38]. The GRADE assessment will be based on our extraction of patient-centered outcomes and a consideration of the methodological quality. Table 2 describes the levels of confidence in the estimates of effect for each outcome, which we will consider to be high, moderate, low, or very low as provided by GRADE criteria [38].

**Table 2:** Four levels of evidence to assess the quality of a body of evidence using the Grading of Recommendations Assessment, Development, and Evaluation (GRADE) approach

Quality level*	Definition
High	We are very confident that the true effect lies close to that of the estimate of the effect
Moderate	We are moderately confident in the effect estimate: The true effect is likely to be close to the estimate of the effect, but there is a possibility that it is substantially different
Low	Our confidence in the effect estimate is limited: The true effect may be substantially different from the estimate of the effect
Very low	We have very little confidence in the effect estimate: The true effect is likely to be substantially different from the estimate of the effect

*Derived from Balshem et al. [38]

According to the GRADE approach, the level of the quality of evidence will be initially high, but the quality can be downgraded for failing to meet the criteria outlined in the five domains of GRADE. We will create GRADE tables for each primary outcome where the level of certainty or strength of evidence and the direction of the treatment effect (in favor of a treatment [positive], no effect, or not in favor of a treatment [negative]). Two reviewers will independently rate the certainty of the evidence from articles. Any disagreements will be discussed until an agreement is reached, involving a third reviewer if needed, to reach a consensus.

## Identification and selection of trials

Results from electronic bibliographic databases will be screened and checked for duplicates by a single reviewer using EndNote (version X9, Clarivate, USA). Before full data extraction, at least two investigators will extract data from identical articles to calibrate and amend the inclusion criteria and extraction if needed. We will repeat calibration phases to ensure that extractions are in accordance with the eligibility criteria and further calibrate the extraction sheet. After each calibration, we will assess agreement in the study design and quality of the data. We will discuss unclear eligibility among the reviewers until consensus (with an overall agreement of Cohen *κ* ≥ 80%) is reached for those cases with unclear eligibility. Any disagreements will be resolved through consensus discussion. We will use a flow chart to present the reasons for the inclusion and exclusion of the articles during the various stages of the systematic review.

## Data collection and extraction

After the calibration period, the remaining trials will be exported to Rayyan [39] as separate files using the RIS export option. Two investigators will then independently determine the eligibility of the trials for inclusion. We will copy and paste the data directly into a Google Excel spreadsheet created for data collection. We would describe the number of studies that have data on each of the characteristics as well as the quality and strength of the evidence. Our variables will comprise extracted data about the baseline characteristics, outcomes of the participants including (patient-centered and disease-specific outcomes), and characteristics of the trials and patients (cohort size, blinding type, phase, intervention and comparator names, study duration, outcomes, adverse events, age, sex, race, and other relevant variables). Missing information will be labeled as such. We aim for an overall agreement in the extracted data of Cohen *κ* ≥ 80% between at least two extractors from a subsample consisting of 10% of the first selected sources. Unclear cases will be discussed until a consensus is reached. The remaining data extraction will be continued by one of the investigators involved in the data extraction from the subsample.

## Data synthesis

For data that may be dichotomous such as the number of patients with at least 75% improvement in pigmentation, we will calculate the odds ratio (OR) and 95% confidence interval (CI). For categorical data, we will extract information about the category assessed, number of patients with a particular outcome, and a number of patients with particular characteristics. We will describe the categories of repigmentation as 75%, > 75%, ≥ 75%, 75 to 90%/100%, or 76 to 90%/100% [1]. We will extract continuous or numerical data as means and standard deviations (SDs), medians and interquartile ranges (IQRs), follow-up, and change from baseline and used to calculate the mean differences with 95% CIs. Reporting of results (mean difference, precision estimates [i.e., 95% CI], and *P* values) from statistical analyses comparing the groups will be extracted.

For adverse events, data for all conditions will be combined. A stratified analysis and meta-regression will be performed to determine whether associations will vary according to treatment type, study design (parallel vs. crossover), repigmentation (categories described above), comparator (active vs. placebo), and duration of follow-up as categories based on a previous systematic review (< 6 months, 6 months to 1 year, < 2 years, ≥ 2 years) [1].

We will group studies with similar indications, treatments, and outcomes. If two or more trials will be within a single grouping, we will pool them using random-effects meta-analysis [40]. Continuous outcomes will be analyzed with the mean difference in change from baseline or the mean difference at follow-up (if the mean difference from change in baseline is not reported or calculations from other data are not possible). Based on a previous study, we will use a single data set instead of more than one to avoid double counting of characteristics [1]. We will select an intervention or dose that is comparable to the other studied interventions or doses in trials with more than one intervention.

We plan to express the measures of the effect of the JAK inhibitors, assess the heterogeneity using the *I*^2^ statistic using meta-analysis techniques (forest plots) if the *I*^2^ statistic is less than 80%, and assess publication bias (if there will be numerous studies about JAK inhibitors) [41]. For studies with similar interventions, we will use a random-effects model to summarize the treatment effect across the studies as a meta-analysis. We will describe data in narrative or descriptive form if it is heterogeneous or unsuitable for pooling (e.g., data only reported in graphs) in tables. Similarly, study characteristics and descriptive information will be displayed in tables. We will perform sensitivity analyses to assess the effect of the trial design. Parallel-group trial results will be included in the primary analysis, while results from other trial designs (factorial or non-parallel designs) will be included in additional analyses. We will present forest plots to display the summarized measures of effect. Funnel plots will be constructed to determine publication bias. If data will be dichotomous, we will use Harbord’s test, while for continuous outcomes we plan to use Egger’s test. *P* values < 0.05 will be considered significant. We propose to use Comprehensive Meta-Analysis version 3.3, Microsoft Excel, and IBM SPSS Statistics for Windows, version 25.0 (IBM Corp., Armonk, NY, USA) or similar programs for the analyses. When the pooled 95% CIs do not cross the line of no effect, we will consider this to indicate statistical significance.

## Discussion

We plan to conduct the proposed systematic review guided by evidence-based guidelines to ensure transparency and replicability of our search. Any deviations from our proposed literature search and strategy, identification and selection of reviews, data collection and extraction, and synthesis of the evidence will be updated in the protocol and described in detail in the final draft of the systematic review. We will additionally collect qualitative evidence, which will provide valuable insights into the nature of data in research studies about vitiligo treatment with JAK inhibitors.

To provide the results of the proposed systematic review to as wide an audience as possible, especially to the lay public and researchers, we plan to publish the results of our review in a peer-reviewed, open-access journal. To disseminate further the results of our systematic review, we plan to present them at international conferences and meetings.

## Limitations

The proposed systematic review may be subject to several limitations. First, although our search strategy will be designed using comprehensive sources, the search may not find all of the trials pertaining to our objective, and review authors may be unavailable for further information.

### Supplementary Information

**Additional file 1. **PRISMA-P (Preferred Reporting Items for Systematic review and Meta-Analysis Protocols) 2015 checklist: recommended items to address in a systematic review protocol.

**Additional file 2. **Draft search strategy from inception to the present for the Web of Science Core Collection, Scopus, and MEDLINE (Ovid) electronic databases.

**Additional file 3. **Pilot search results of the Web of Science Core Collection, Scopus, and MEDLINE (Ovid) electronic databases from inception to 27/01/2024.

## Data Availability

This proposed protocol is available at PROSPERO (CRD42023383920).

## Abbreviations

CO2: Carbon dioxide
DLQI: Dermatology Quality of Life Index
JAK: Janus kinase
PRISMA-P: Preferred Reporting Items for Systematic Reviews and Meta-Analyses Protocols
RCT: Randomized controlled trials
RoB: Risk of bias
UVA: Ultraviolet A
VitiQoL: Vitiligo Quality of Life Index
