# Supplementary material for: Protocol for a systematic review and meta-analysis on Janus kinase inhibitors in the management of vitiligo
Source: Syst Rev. 2024 Apr 19;13:110. doi: 10.1186/s13643-024-02522-3 (PMC11027385; doi:10.1186/s13643-024-02522-3)
Supplement: Supplementary file 2 — Additional file 2. Draft search strategy from inception to the present for the Web of Science Core Collection, Scopus, and MEDLINE (Ovid) electronic databases. [file 13643_2024_2522_MOESM2_ESM.docx]

Additional File 2. Draft search strategy from inception to 08/02/2023 for the Web of Science Core Collection, Scopus, and MEDLINE (Ovid) electronic databases.

Keywords: vitiligo, JAK, baricitinib, baricinix, tofacitinib, inhibitor, ruxolitinib, olumiant, Xeljanz, Janus kinase, Olumiant, Jakafi, Rinvoq, Inrebic, Cibinqo, Jyseleca, Opzelura

Web of Science Core Collection

ab=(vitiligo) and ab=((JAK) or(baricitinib) or(baricinix) or(tofacitinib)or (inhibitor) or(ruxolitinib)or(olumiant)or(Xeljanz) or(Janus kinase) or(Olumiant)or( Jakafi )or(Rinvoq)or(Inrebic)or(Cibinqo) or(Jyseleca)or(Opzelura))

Scopus

( ( KEY ( janus AND kinase AND inhibitors ) ) OR ( TITLE-ABS ( janus W/3 kinas$ W/9 inhibit$ ) OR TITLE-ABS ( jak W/3 kinas$ W/9 inhib$ ) OR TITLE-ABS ( jak W/9 inhibitor$ ) OR TITLE-ABS ( baricitinib OR barinat OR baricinix OR tofacitinib OR ruxolitinib OR olumiant OR xeljanz OR jakafi OR opzelura ) ) ) AND ( ( ( KEY ( vitiligo ) ) OR ( ( KEY ( hypopigmentation ) ) AND NOT ( KEY ( albinism ) ) ) ) OR ( TITLE-ABS ( vitiligo OR vitiliqo OR vitiligin$ OR vitiligoj$ OR bielactwo OR leucoderm$ OR leukoderm$ ) ) OR ( TITLE-ABS ( piebald W/3 skin ) OR TITLE-ABS ( white W/3 patch$ W/9 skin ) OR TITLE-ABS ( depigment$ W/3 skin ) OR TITLE-ABS ( hypopigment$ W/3 skin ) ) )

MEDLINE (Ovid)

1 exp Vitiligo/ (6230)

2 exp Hypopigmentation/ (11549)

3 exp Albinism/ (4976)

4 2 not 3 (7763)

5 (vitiligo or vitiliqo or vitiligin$ or vitiligoj$ or bielactwo or leucoderm$ or leukoderm$).tw. (8831)

6 (piebald adj3 skin).tw. (8)

7 (white adj3 patch$ adj9 skin).tw. (89)

8 (depigment$ adj3 skin).tw. (760)

9 (hypopigment$ adj3 skin).tw. (516)

10 1 or 4 or 5 or 6 or 7 or 8 or 9 (11614)

11 exp Janus Kinase Inhibitors/ (1258)

12 (janus adj3 kinas$ adj9 inhibit$).tw. (3644)

13 (jak adj3 kinas$ adj9 inhib$).tw. (1349)

14 (jak adj9 inhibitor$).tw. (3936)

15 (baricitinib or barinat or baricinix or tofacitinib or ruxolitinib or olumiant or Xeljanz or Jakafi or Opzelura).tw. (4860)

16 11 or 12 or 13 or 14 or 15 (9411)

17 16 and 10 (101)

18 comment.pt. (989370)

19 review.pt. (3085299)

20 English abstract.pt. (1470272)

21 17 not 18 (99)

22 21 not 19 (48)

23 22 not 20 (48)
