# Supplementary material for: Protocol for a systematic review and meta-analysis on Janus kinase inhibitors in the management of vitiligo
Source: Syst Rev. 2024 Apr 19;13:110. doi: 10.1186/s13643-024-02522-3 (PMC11027385; doi:10.1186/s13643-024-02522-3)
Supplement: Supplementary file 3 — Additional file 3. Pilot search results of the Web of Science Core Collection, Scopus, and MEDLINE (Ovid) electronic databases from inception to 27/01/2024. [file 13643_2024_2522_MOESM3_ESM.docx]

Additional File 3. Pilot search results of the Web of Science Core Collection, Scopus, and MEDLINE (Ovid), electronic databases from inception to 27/01/2024.

Keywords: vitiligo, JAK, baricitinib, baricinix, tofacitinib, inhibitor, ruxolitinib, olumiant, Xeljanz, Janus kinase, Olumiant, Jakafi, Rinvoq, Inrebic, Cibinqo, Jyseleca, Opzelura, ritlecitinib, delgocitinib, upadacitinib, brepocitinib, ifidancitinib, cerdulatinib

| Web of Science Core Collection = 491 | ab=(vitiligo) and ab=((JAK) or(baricitinib) or(baricinix) or(tofacitinib)or (inhibitor) or(ruxolitinib)or(olumiant)or(Xeljanz) or(Janus kinase) or(Olumiant)or( Jakafi )or(Rinvoq)or(Inrebic)or(Cibinqo) or(Jyseleca)or(Opzelura) or (ritlecitinib), (delgocitinib), (upadacitinib), (brepocitinib), (ifidancitinib), (cerdulatinib)) |
| --- | --- |
| Scopus = 294 | ( ( KEY ( janus AND kinase AND inhibitors ) ) OR ( TITLE-ABS ( janus W/3 kinas$ W/9 inhibit$ ) OR TITLE-ABS ( jak W/3 kinas$ W/9 inhib$ ) OR TITLE-ABS ( jak W/9 inhibitor$ ) OR TITLE-ABS ( baricitinib OR barinat OR baricinix OR tofacitinib OR ruxolitinib OR olumiant OR xeljanz OR jakafi OR opzelura OR ritlecitinib OR delgocitinib OR upadacitinib OR brepocitinib OR ifidancitinib OR cerdulatinib ) ) ) AND ( ( ( KEY ( vitiligo ) ) OR ( ( KEY ( hypopigmentation ) ) AND NOT ( KEY ( albinism ) ) ) ) OR ( TITLE-ABS ( vitiligo OR vitiliqo OR vitiligin$ OR vitiligoj$ OR bielactwo OR leucoderm$ OR leukoderm$ ) ) OR ( TITLE-ABS ( piebald W/3 skin ) OR TITLE-ABS ( white W/3 patch$ W/9 skin ) OR TITLE-ABS ( depigment$ W/3 skin ) OR TITLE-ABS ( hypopigment$ W/3 skin ) ) ) |
| MEDLINE (Ovid) = 83 | **1**  exp Vitiligo/ (6566)  **2**  exp Hypopigmentation/ (11988)  **3**  exp Albinism/ (5057)  **4**  2 not 3 (8134)  **5**  (vitiligo or vitiliqo or vitiligin$ or vitiligoj$ or bielactwo or leucoderm$ or leukoderm$).tw. (9483)  **6**  (piebald adj3 skin).tw. (9)  **7**  (white adj3 patch$ adj9 skin).tw. (100)  **8**  (depigment$ adj3 skin).tw. (824)  **9**  (hypopigment$ adj3 skin).tw. (539)  **10**  1 or 4 or 5 or 6 or 7 or 8 or 9 (12333)  **11**  exp Janus Kinase Inhibitors/ (1738)  **12**  (janus adj3 kinas$ adj9 inhibit$).tw. (4555)  **13**  (jak adj3 kinas$ adj9 inhib$).tw. (1679)  **14**  (jak adj9 inhibitor$).tw. (4774)  **15**  (baricitinib or barinat or baricinix or tofacitinib or ruxolitinib or olumiant or Xeljanz or Jakafi or Opzelura or ritlecitinib or delgocitinib or upadacitinib or brepocitinib or ifidancitinib or cerdulatinib).tw. (6683)  **16**  11 or 12 or 13 or 14 or 15 (11825)  **17**  10 and 16 (158)  **18**  comment.pt. (1029833)  **19**  review.pt. (3270697)  **20**  English abstract.pt. (1497777)  **21**  17 not 18 (154)  **22**  21 not 19 (84)  **23**  22 not 20 (83) |
